# Phenomapping the Response of Patients With Ischemic Cardiomyopathy With Reduced Ejection Fraction to Surgical Revascularization

**DOI:** 10.1002/clc.70094

**Published:** 2025-02-03

**Authors:** Tejus Satish, Nicholas S. Hendren, Matthias Peltz, Christopher A. Heid, Maryjane Farr, Anthony Bavry, Saket Girotra, Dharam J. Kumbhani, Mark H. Drazner, W. H. Wilson Tang, Justin L. Grodin

**Affiliations:** ^1^ Department of Internal Medicine, Division of Cardiology University of Texas Southwestern Medical Center Dallas Texas USA; ^2^ Department of Cardiovascular Medicine Cleveland Clinic Cleveland Ohio USA

**Keywords:** clustering, coronary artery bypass grafting, heart failure with reduced ejection fraction, machine learning

## Abstract

**Background:**

Coronary artery bypass grafting (CABG) has demonstrated long‐term mortality benefits in patients with HFREF and obstructive coronary artery disease (CAD), but whether phenotypic heterogeneity influences the benefits of CABG is unknown. We applied clustering analysis to STICHES (Surgical Treatment for Ischemic Heart Failure Extension Study) to identify phenogroups with different long‐term risk profiles and investigate differences in CABG benefits between phenogroups.

**Methods and Results:**

STICHES was a randomized controlled trial evaluating the effect of CABG in addition to medical therapy versus medical therapy alone. We split the STICHES participants into derivation (*n* = 753) and validation (*n* = 459) cohorts. We phenomapped the derivation cohort using penalized model‐based clustering. We fit multivariable Cox models to investigate long‐term differences in all‐cause mortality, cardiovascular (CV) mortality, and a composite of all‐cause mortality/CV hospitalization between phenogroups and whether phenogroup assignment modified the effects of CABG on these outcomes. Findings were internally validated on the validation cohort. Four phenogroups were identified in the derivation cohort. The highest‐risk group was at a twofold greater risk of death (HR: 2.0, 95% CI: 1.4–2.9, *p* < 0.001) and CV death (HR: 2.0, 95% CI: 1.3–3.1, *p* = 0.002), and a 1.5‐fold greater risk for death/CV hospitalization (HR: 1.5, 95% CI: 1.1–2.1, *p* = 0.016). Phenogroup assignment did not modify the effects of CABG on the outcomes (*p* > 0.05 for all). Similar results were obtained in the validation cohort.

**Conclusions:**

The beneficial effects of CABG on all‐cause mortality, CV mortality, and a composite of all‐cause mortality and CV hospitalization persist despite phenotypic heterogeneity in HFREF and CAD.

## Graphical abstract

a) Kaplan‐Meier curve for all‐cause mortality in the derivation cohort stratified by clustering‐derived phenogroups. P‐value in the lower left of a) is the result of the log‐rank test. b) Hazard ratios for all‐cause mortality as obtained by multivariable Cox proportional‐hazards modeling in the derivation cohort stratified by clustering‐derived phenogroups. Similar results were obtained in the validation cohort. Note that the P‐value reported for the interaction effect between treatment assignment and phenogroup assignment is the result of the likelihood‐ratio test for the addition of an interaction term between phenogroup assignment and treatment assignment to the Cox proportional‐hazards model.

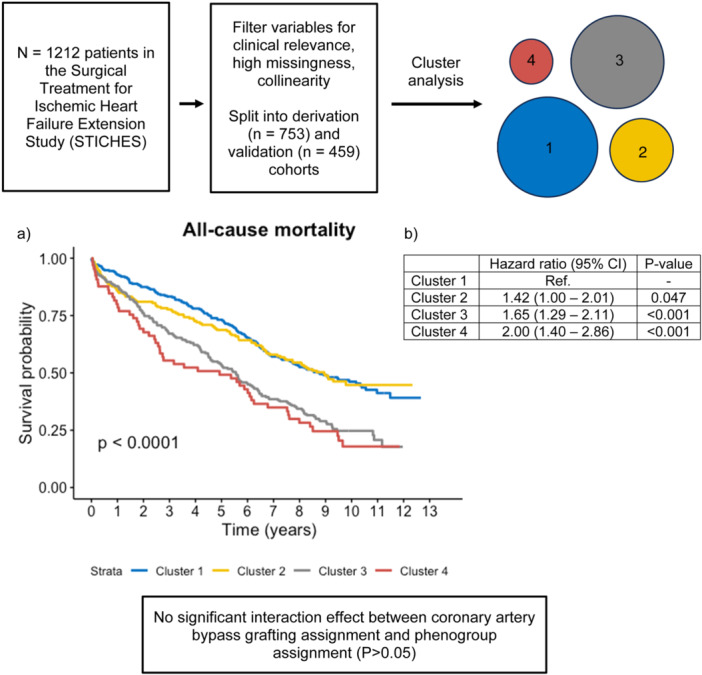

**Abbreviations**
AF: atrial fibrillation/flutter
BUN: blood urea nitrogen
CABG: coronary artery bypass grafting
CAD: coronary artery disease
CKD: chronic kidney disease
CV: cardiovascular
HFREF: Heart failure with reduced ejection fraction
LVEF: left ventricular ejection fraction
MCS‐12: mental component score‐12
PCS‐12: physical component score‐12

## 1 Introduction

Heart failure with reduced left ventricular ejection fraction (HFREF) is a heterogenous disease with a wide range of phenotypic variability and risk profiles which may influence response to specific treatments [1, 2, 3]. Cardiomyopathy due to obstructive coronary artery disease (CAD) represents ~50% of all cases of HFREF and, when appropriate, benefits from surgical revascularization [4]. Surgical revascularization via coronary artery bypass grafting (CABG) has demonstrated long‐term mortality benefits in patients with ischemic HFREF [5]. Whether phenotypic heterogeneity among populations with HFREF and obstructive CAD influences the prognostic benefit of CABG is not well known. We hypothesize that clinical characteristics will differentiate patients with HFREF and obstructive CAD into novel phenogroups of patients, and that these phenogroups will have different prognoses and treatment responses to CABG. Leveraging data from the Surgical Treatment for Ischemic Heart Failure Extension Study (STICHES), we sought to apply cluster analysis methods to identify novel phenogroups, determine their prognostic profiles, and evaluate for differential prognostic benefits to CABG between phenogroups.

## 2 Materials and Methods

### 2.1 Study Population

This analysis was performed on publicly available deidentified data from the STICH trial and its extension, STICHES, available on the National Heart, Lung, and Blood Institute's Biologic Specimen and Data Repository Information Coordinating Center. Institutional Review Board approval was waived due to the use of publicly available deidentified data. The protocol, design, and analysis of STICH/STICHES have been previously reported [5]. Briefly, the STICH trial was conducted between July 2002 and May 2007 and included 1212 patients across 127 clinical sites in 26 countries. Included patients were > 18 years of age, had a left‐ventricular ejection fraction (LVEF) of 35% or less within 3 months of trial entry, and had coronary artery disease (CAD) suitable for revascularization. STICH trial follow‐up was extended to a median of 9.8 years in STICHES. STICH employed a stratified randomization structure to test two interventions: the effect of coronary artery bypass grafting (CABG) and the effect of surgical ventricular reconstruction. Eligibility criteria for the study arms and procedures have been previously reported [5]. Participants eligible for the arm testing the effect of CABG relevant to this study were placed into two strata, A and B, depending on their eligibility for surgical ventricular reconstruction. Participants in the A and B strata not randomized to receive surgical ventricular reconstruction were randomized 1:1 to receive either guideline‐directed medical therapy alone or guideline‐directed medical therapy plus CABG.

### 2.2 Clustering Procedure

STICH data dictionaries were examined to select 79 clinically relevant variables including demographics, physical measurements, laboratory measurements, past medical history, medications, echocardiographic data, exercise tolerance, cardiac viability data, cardiac biomarkers, and left heart catheterization results. Fifteen variables with greater than 30% missingness were excluded. Pearson's r coefficient, Spearman's rank correlation coefficient, point‐biserial coefficients, and Cramér's V were calculated as appropriate to identify highly collinear variable pairs with a correlation coefficient > 0.6. The variable in each pair deemed less clinically relevant was discarded. Following these steps, 58 variables remained. The R *MICE* package was used to impute missing values via multiple imputation using chained equations with 30 iterations. The imputed data were randomly split 60:40 into a primary data set and an internal validation data set (*n* = 753 in training set, *n* = 459 in internal validation set). Model‐based clustering, as described previously and implemented in the R package *VarSelLCM*, was utilized to derive clusters in the training set [6]. Briefly, model‐based clustering is a mixture‐modelling method well‐suited for mixed‐type data (i.e., data containing continuous, categorical, and ordinal variables). This method utilizes Gaussian copulas to join the appropriate one‐dimensional marginal cumulative distribution functions for each variable type into a cumulative distribution function for each cluster. Model‐based clustering was applied to fit models containing between 2 and 6 clusters to the data. Each clustering model optimization was run 6000 times with varying initialization conditions to ensure model stability. The optimal number of clusters was determined via maximization of the Bayesian Information Criterion. Student's t‐tests, analysis of variance, Kruskal‐Wallis tests, chi‐squared tests, and Fisher's exact tests were applied as appropriate to investigate differences in characteristics between derived clusters.

### 2.3 Cluster Characterization and Outcomes Analysis

The outcomes of interest for the present analysis were the primary and secondary outcomes of the STICHES trial. The primary outcome was all‐cause mortality, and secondary outcomes were cardiovascular (CV) mortality and a composite outcome of all‐cause mortality and CV hospitalization. CV death and CV hospitalizations were adjudicated by the clinical endpoint adjudication committee of the STICHES trial. Kaplan‐Meier curves and log‐rank tests were computed for right‐censored time‐to‐event data for the selected outcomes in the training data set. Multivariable Cox proportional‐hazards models adjusted for treatment assignment, randomization strata, age, sex, race, New York Heart Association (NYHA) class, history of myocardial infarction, history of CABG, history of percutaneous coronary intervention, number of vessels with > 75% stenosis, LVEF, and enrollment region were used to determine outcome differences between clusters. An identical analysis was performed on the internal validation data set to verify any discriminative effect of cluster assignment on outcomes was replicable. An interaction term between treatment assignment and cluster membership was added to the Cox models for each outcome, and a likelihood ratio test was run to determine whether this addition significantly improved model fit. In all Cox models, the proportional hazards assumption was verified via visual inspection of log‐log plots and scaled Schoenfeld residuals. A secondary analysis using the Bonferroni‐adjusted Kruskal‐Wallis test was performed to investigate whether short‐term outcomes measured in the STICHES trial (Postsurgical hospital stay length, Postsurgical hospitalization length greater than 30 days, death within 30 days post‐surgery, death or continued hospitalization at 30 days post‐surgery, MI within 30 days post‐surgery, stroke within 30 days post‐surgery) differed between clusters. Twelve‐item Short Form Survey physical (PCS‐12) and mental (MCS‐12) component scores were calculated for all participants. Scores between clusters at various intervals were compared using ANOVA with the Bonferroni correction, and scores between treatments within clusters at various intervals were compared using t‐tests with the Bonferroni correction. Two‐sided *P*‐values < 0.05 were considered statistically significant unless otherwise noted. All analyses were carried out in R version 4.1.1.

## 3 Results

### 3.1 Phenogroup Derivation and Characteristics

A list of all model variables is presented in Table S1. In both the derivation and validation cohorts, a four‐cluster model produced the best fit to the data as determined by the highest Bayesian Information Criterion (Figure S1). Measures of diastolic function (E/A ratio, mitral valve E velocity) and kidney function (creatinine, blood urea nitrogen (BUN), history of chronic kidney disease) were highly discriminatory (Figure S2). Baseline clinical characteristics of each phenogroup are presented and compared in Table 1. All phenogroups predominantly contained male patients with NYHA class 2 and 3 heart failure with similar use of heart failure pharmacotherapies. Within phenogroups, baseline clinical characteristics did not differ by treatment assignment (Table S2).

**Table 1:** Baseline clinical characteristics.

	Cluster 1	Cluster 2	Cluster 3	Cluster 4	*p*‐value
n	287	179	222	65	—
**Patient characteristics**					
Mean age [years] (SD)*	60.7 (8.8)	57.6 (8.4)	60.0 (8.8)	64.9 (10.4)	< 0.001
Female sex (%)*	42 (14.6)	27 (15.1)	9 (4.1)	12 (18.5)	< 0.001
Race (%)*					< 0.001
White	226 (78.7)	66 (36.9)	158 (71.2)	51 (78.5)	—
Asian	8 (2.8)	109 (60.9)	11 (5.0)	7 (10.8)	—
Black	8 (2.8)	3 (1.7)	5 (2.3)	4 (6.2)	—
Other	45 (15.7)	1 (0.6)	48 (21.6)	3 (4.6)	—
Region (%)					< 0.001
North America	49 (17.1)	26 (14.6)	55 (24.8)	36 (55.3)	—
Europe	211 (73.5)	38 (21.2)	135 (60.8)	18 (27.7)	—
Asia‐Pacific	14 (4.9)	108 (60.3)	19 (8.6)	8 (12.3)	—
South America	13 (4.5)	7 (3.9)	13 (5.9)	3 (4.6)	—
**Medical history**					
Myocardial infarction (%)*	240 (83.6)	118 (65.9)	175 (78.8)	53 (81.5)	< 0.001
Percutaneous coronary intervention (%)*	33 (11.5)	24 (13.4)	36 (16.2)	10 (15.4)	0.451
Coronary artery bypass graft (%)*	11 (3.8)	1 (0.6)	11 (5.0)	4 (6.2)	0.026
Implantable cardioverter‐defibrillator (%)*	1 (0.3)	5 (2.8)	10 (4.5)	7 (10.8)	< 0.001
Diabetes (%)*	87 (30.3)	97 (54.2)	73 (32.9)	50 (76.9)	< 0.001
Hypertension (%)*	191 (66.6)	95 (53.1)	123 (55.4)	46 (70.8)	0.0032
Hyperlipidemia (%)*	195 (67.9)	67 (37.6)	142 (64.3)	44 (67.7)	< 0.001
Atrial flutter/atrial fibrillation (%)*	12 (4.2)	10 (5.6)	56 (25.2)	13 (20.0)	< 0.001
Chronic kidney disease (%)*	10 (3.5)	5 (2.8)	4 (1.8)	42 (64.6)	< 0.001
Active smoker (%)*	63 (22.0)	33 (18.4)	62 (27.9)	9 (13.8)	0.043
**Clinical characteristics**					
NYHA stage 3+ (%)* ^,^ a	94 (32.8)	47 (26.3)	104 (46.9)	36 (55.4)	< 0.001
Mean pulse [bpm] (SD)*	70.9 (10.7)	78.9 (10.7)	76.0 (14.1)	79.8 (27.5)	< 0.001
Mean systolic blood pressure [mmHg] (SD)*	124.2 (15.1)	122.9 (19.1)	117.0 (15.7)	118.1 (18.8)	< 0.001
Mean diastolic blood pressure [mmHg] (SD)	76.9 (10.0)	77.3 (11.1)	74.6 (9.6)	68.5 (13.5)	< 0.001
Mean body mass index (SD)*	28.5 (4.5)	24.8 (3.8)	27.1 (3.8)	28.7 (6.3)	< 0.001
6‐min walk test distance [m] (SD)*	342.5 (107.2)	325.8 (124.0)	345.1 (124.6)	256.2 (114.5)	< 0.001
**Baseline medications**					
Aspirin (%)*	250 (87.1)	151 (84.4)	175 (78.8)	60 (92.3)	0.021
Clopidogrel (%)*	23 (8.0)	78 (43.6)	22 (9.9)	14 (21.5)	< 0.001
Statin (%)*	229 (79.8)	161 (89.9)	173 (77.9)	50 (76.9)	0.0047
Angiotensin‐converting enzyme inhibitor (%)*	239 (83.3)	145 (81.0)	190 (85.6)	43 (66.2)	0.007
Angiotensin receptor blocker (%)*	24 (8.4)	18 (10.1)	19 (8.6)	12 (18.5)	0.110
Beta‐blocker (%)*	260 (90.6)	146 (81.6)	191 (86.0)	55 (84.6)	0.039
K‐wasting diuretic (%)*	175 (61.0)	116 (64.8)	152 (68.8)	58 (89.2)	< 0.001
K‐sparing diuretic (%)*	127 (44.3)	80 (44.7)	123 (55.4)	26 (40.0)	0.032
Digoxin (%)*	33 (11.5)	49 (27.4)	48 (21.6)	25 (38.5)	< 0.001
Nitrate (%)*	161 (56.1)	113 (63.1)	90 (40.7)	32 (49.2)	< 0.001
Warfarin (%)*	21 (7.3)	9 (5.0)	34 (15.3)	10 (15.4)	< 0.001
Amiodarone (%)*	20 (7.0)	18 (10.1)	24 (10.8)	13 (20.0)	0.021
**Laboratory measurements**					
Mean sodium [mEq/L] (SD)*	140.0 (3.0)	137.9 (5.7)	140.0 (3.0)	137.0 (4.2)	< 0.001
Mean creatinine [mg/dL] (SD)*	1.1 (0.2)	1.1 (0.2)	1.1 (0.2)	2.3 (2.1)	< 0.001
Mean blood urea nitrogen [mg/dL] (SD)*	28.7 (18.4)	21.2 (10.1)	32.8 (19.1)	56.0 (43.4)	< 0.001
Mean hemoglobin [g/dL] (SD)*	14.2 (1.5)	13.0 (1.8)	14.1 (1.6)	12.2 (1.7)	< 0.001

*Note:* Baseline clinical characteristics of patients in the STICHES trial stratified by model‐based clustering derived phenogroup.

Abbreviations: CCS, Canadian Cardiovascular Society; NYHA, New York Heart Association.

* Variable was part of final mixture model after missingness and collinearity check steps.

^a^ In full model, NYHA class was an ordinal variable with levels 1, 2, 3, 4.

**Figure 1:**
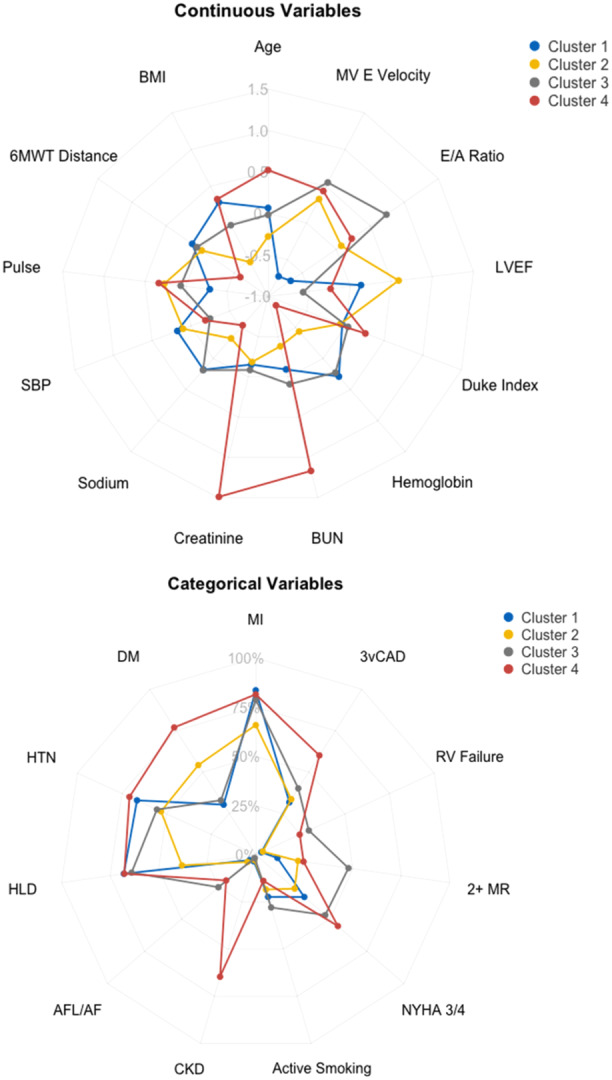
Clinical charateristics of derived phenogroups. Presented are radar plots of relevant clinical characteristics of phenogroups derived via model‐based clustering of the STICHES cohort. Continuous variables were Z‐normalized to the full cohort. RV (right ventricular) failure was defined as moderate‐severe RV dysfunction on echocardiography. 6MWT, 6‐min walk test; AFL/AF, atrial flutter/fibrillation; BMI, body mass index; BUN, blood urea nitrogen; CAD, coronary artery disease; CKD, chronic kidney disease; DM, diabetes mellitus; HLD, hyperlipidemia; HTN, hypertension; LVEF, left ventricular ejection fraction; MI, myocardial infarction; MR, mitral regurgitation; MV, mitral valve; NYHA, New York Heart Association; RV, right ventricle; SBP, systolic blood pressure.

A radar plot visually summarizing selected phenogroup characteristics is presented in Figure 1. Phenogroup 1 contained mostly White patients from the Europe region exhibiting comparatively low rates of diabetes, chronic kidney disease (CKD), and atrial fibrillation/flutter (AF) relative to the other groups. Phenogroup 2 had the lowest average age and body mass index (BMI) and was a mixture of White and Asian patients in the Asia‐Pacific and Europe regions with comparatively low rates of prior MI, CKD, and AF and intermediate rates of diabetes and smoking relative to the other groups. Phenogroup 3 predominantly contained White patients from the Europe region with comparatively low rates of diabetes and high rates of AF and smoking relative to the other groups. Phenogroup 4 had the highest average age and contained mostly White patients across the USA, Canada, and Europe regions with the lowest exercise tolerance by the 6‐min walk test. Relative to the other groups, Phenogroup 4 had higher rates of diabetes, AF, CKD, and depression. Phenogroup 4 also had the lowest average hemoglobin and highest average serum creatinine and BUN.

Echocardiographic and coronary angiographic data of each phenogroup are presented in Online Table S3. The degree of CAD as measured by the Duke CAD index was relatively similar across phenogroups; however, the distribution of angiographically significant CAD between vessels and prevalence of three‐vessel disease differed significantly between phenogroups. Phenogroups 1 and 2 had relatively higher LVEF compared with phenogroups 3 and 4. There was a progressive increase in E/e’ ratio from phenogroup 1 to phenogroup 4. There were no significant differences in intraventricular septum or posterior wall thickness measurements between phenogroups. Phenogroups 3 and 4 displayed higher E/A ratios than phenogroups 1 and 2, with phenogroup 3 having the overall highest E/A ratio. Phenogroup 3 displayed the highest proportion of patients with moderate‐severe mitral regurgitation and the highest proportion of patients with moderate‐severe right ventricular dysfunction.

### 3.2 Outcome Analysis on Derived Phenogroups

**Figure 2:**
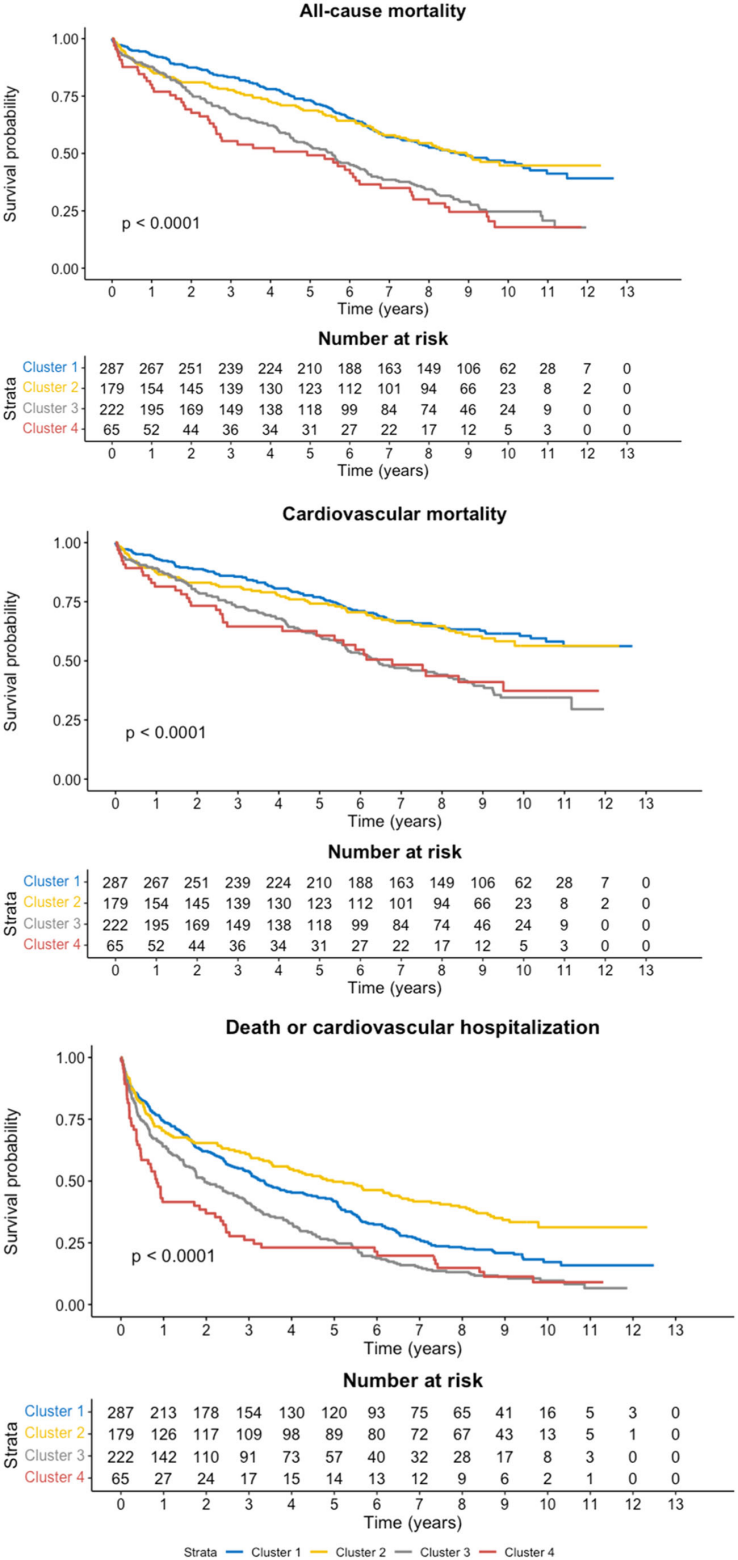
Kaplan–Meier curves for study outcomes stratified by derived phenogroup. Kaplan–Meier curves of key outcomes in the STICHES trial stratified by model‐based clustering derived phenogroups. *P*‐values in the lower left are the result of the log‐rank test.

Cumulative incidence rates of primary and secondary outcomes stratified by phenogroup are presented in Table S4. In the derivation cohort (*n* = 753), over 4,751 person‐years of follow‐up, 466 (61.9%) patients died, 337 (44.8%) patients experienced CV death, and 608 (80.7%) patients experienced a combined outcome of death and CV hospitalization. Kaplan‐Meier curves for the primary and secondary outcomes are presented in Figure 2, with results of the accompanying multivariable Cox models presented in Table 2. Relative to phenogroup 1, phenogroups 2, 3, and 4 exhibited successively greater statistically‐significant increases in the risk of all‐cause mortality. A similarly consistent increase in the risk of CV mortality and the risk of a composite of all‐cause mortality and CV hospitalization from phenogroup 1 to 4 was observed. However, phenogroup 2 did not have a significantly higher risk of these outcomes compared with phenogroup 1, while phenogroups 3 and 4 did. Postsurgical short‐term outcomes stratified by phenogroup are presented in Online Table S5. There were no significant differences in any short‐term outcomes between the groups (Bonferroni‐adjusted Kruskal–Wallis test, alpha = 0.01).

**Table 2:** Hazard ratios of key outcomes.

	Hazard ratio (95% CI)	*p*‐value
**All‐cause mortality**		
Cluster 1	Ref.	—
Cluster 2	1.42 (1.00–2.01)	0.047
Cluster 3	1.65 (1.29–2.11)	< 0.001
Cluster 4	2.00 (1.40–2.86)	< 0.001
**Cardiovascular mortality**		
Cluster 1	Ref.	—
Cluster 2	1.32 (0.86–2.01)	0.20
Cluster 3	1.69 (1.27–2.25)	< 0.001
Cluster 4	1.99 (1.27–3.07)	0.0020
**Death or cardiovascular hospitalization**		
Cluster 1	Ref.	—
Cluster 2	0.83 (0.61–1.12)	0.21
Cluster 3	1.36 (1.10–1.68)	0.0052
Cluster 4	1.49 (1.08–2.07)	0.016

*Note:* Hazard ratios of key outcomes in the STICHES trial as obtained by multivariable Cox proportional‐hazards modelling stratified by model‐based clustering derived phenogroups.

### 3.3 Internal Model Validation

Cumulative incidence rates of primary and secondary outcomes stratified by phenogroup for the internal validation cohort (*n* = 459) are presented in Online Table S6. Rates of all outcomes were relatively similar in the validation cohort as compared with the derivation cohort, with 291 (63.4%) patients dying, 207 (45.1%) patients experiencing CV death, and 383 (83.4%) patients experiencing a composite outcome of death and CV hospitalization. Kaplan‐Meier curves for the primary and secondary outcomes are presented in Figure S3, with results of the accompanying multivariable Cox models presented in Online Table S7. Consecutively greater risks for all outcomes were seen in phenogroups 2, 3, and 4 versus phenogroup 1. This effect was statistically significant for phenogroups 3 and 4 for all‐cause and CV mortality, and only for phenogroup 4 for the composite of death and CV hospitalization.

### 3.4 Effect of Coronary Artery Bypass Grafting Between Phenogroups

**Figure 3:**
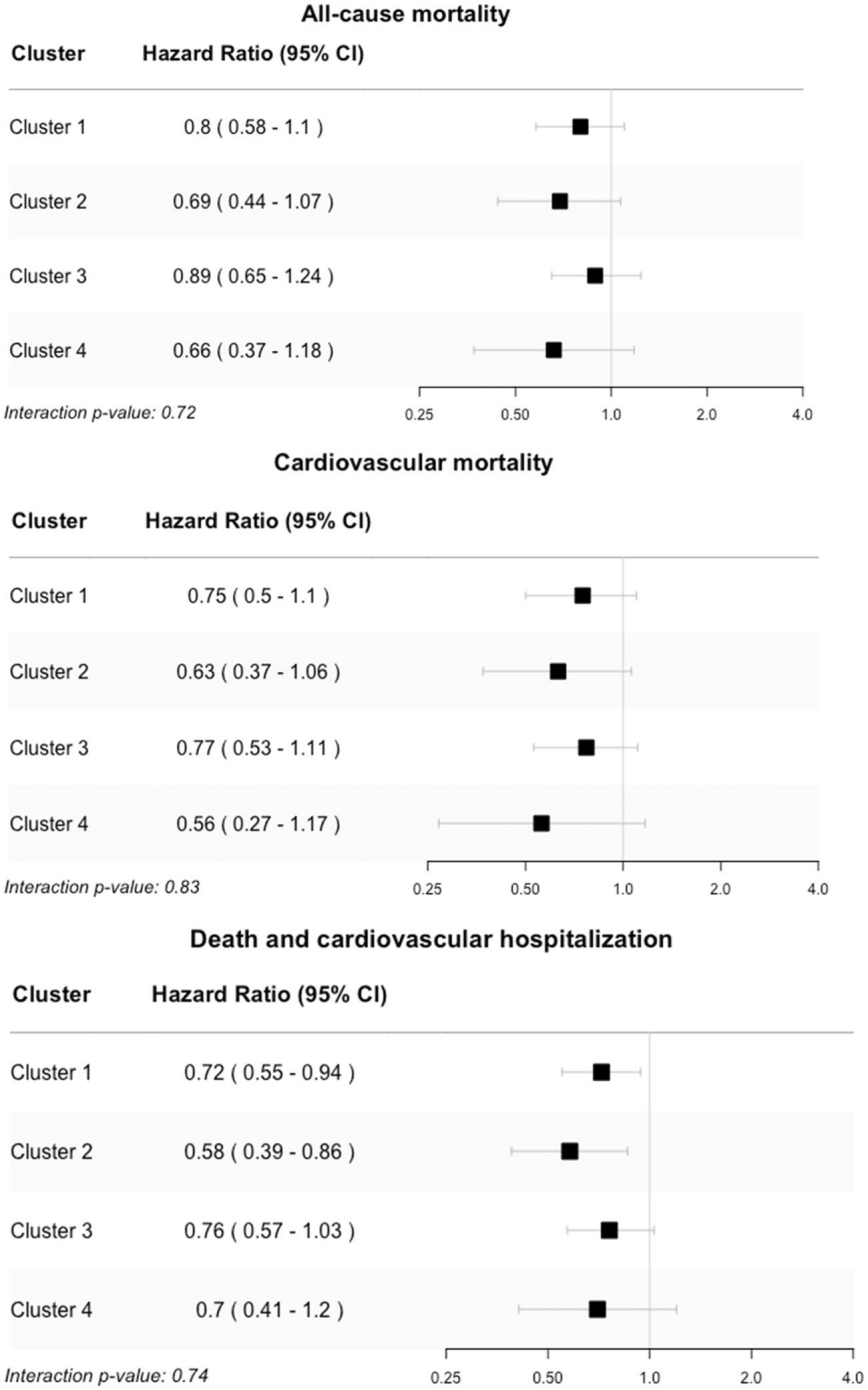
Impact of phenogroup assignment on coronary artery bypass grafting effects. Effect of coronary artery bypass grafting (CABG) in addition to optimal medical therapy versus optimal medical therapy alone on key outcomes in the STICHES trial stratified by model‐based clustering derived phenogroups. *P*‐values shown in the lower left are from a likelihood‐ratio test for the addition of an interaction term between phenogroup assignment and treatment assignment to the Cox proportional‐hazards model for each outcome.

Within‐phenogroup treatment effects of CABG in addition to optimal medical therapy versus optimal medical therapy alone are presented in Figure 3. While the risk of the primary and secondary outcomes was lower in the surgical group in each phenogroup, these effects did not reach statistical significance with the exception of in phenogroups 1 and 2 for the composite of death and CV hospitalization. For all outcomes, there was no significant interaction effect between treatment assignment and phenogroup assignment.

### 3.5 Quality‐of‐Life Differences Between Phenogroups

SF‐12 physical component (PCS‐12) and mental component (MCS‐12) scores for each phenogroup are presented in Online Figure S4. PCS‐12 scores differed between the phenogroups, with phenogroup 2 displaying the highest scores and phenogroup 4 displaying the lowest scores (Bonferroni‐adjusted ANOVA, alpha = 0.01). MCS‐12 scores did not differ significantly between phenogroups (Bonferroni‐adjusted ANOVA, alpha = 0.01). Across all phenogroups, PCS‐12 and MCS‐12 scores increased from baseline to the first reassessment at 4 months. PCS‐12 and MCS‐12 scores between the optimal heart failure therapy and optimal heart failure therapy with CABG arms in each phenogroup are presented in Figure S5. There were no significant score differences between treatment groups in any phenogroup (Bonferroni‐adjusted *t*‐test, alpha = 0.0025).

## 4 Discussion

This post‐hoc analysis of STICHES applied model‐based clustering to derive phenogroups with distinct clinical and echocardiographic characteristics that stratified different risk and clinical trajectory profiles. The phenogroups were internally validated on a randomly selected subset of the STICHES data set. These phenogroups differed in their longitudinal risks of the primary outcome (all‐cause mortality) and of the secondary outcomes (CV mortality and a composite of death and CV hospitalization). However, these phenogroups did not modify the impact of CABG versus medical therapy on long‐term outcomes. These observations highlight the clinical heterogeneity present in populations with obstructive CAD and HFREF and how that may influence long‐term risk, but also serve to inform that this heterogeneity does not impact the benefit of CABG.

While HFrEF management guidelines are typically constructed using distinct phenotypes (e.g., ischemic cardiomyopathy vs. nonischemic cardiomyopathy, left‐sided vs. right‐sided) and discrete risk factors (e.g., diabetes, kidney disease, atrial fibrillation), these phenotypes and risk factors frequently overlap [7, 8]. This clinical interdependence has driven prior attempts to apply various advanced statistical learning methods, including cluster analysis, to dense phenotypic data from cohorts of patients with HFrEF to identify complex phenogroups to inform prognostication and treatment [3, 8, 9]. The present analysis utilized this paradigm to query whether CABG provides differential benefits to phenogroups of patients at different longitudinal clinical risk. While a variety of well‐defined approaches have been previously used in clustering analyses of patients with heart failure (e.g., hierarchical clustering, k‐means clustering, k‐medoids clustering, and latent class analysis), many such approaches are limited by their inability to handle mixed data containing continuous, categorical, and ordinal elements [3, 9, 10, 11, 12, 13]. Thus, we elected to use a relatively novel implementation of Gaussian copula‐based mixture‐modelling uniquely well‐suited to the clustering of high‐dimensional mixed data [6] that has been used in prior heart failure datasets [14].

In pre‐specified and post‐hoc subgroup analyses of the STICHES trial, no risk factors were observed to be associated with a lower long‐term benefit of CABG over medical treatment alone. However, other studies investigating the benefit of early invasive management of ischemic heart disease (either via CABG or percutaneous coronary intervention) versus medical therapy alone with different inclusion criteria and design strategies have produced conflicting results (albeit generally with a trend towards the superiority of invasive therapy) [5, 15, 16, 17, 18]. Given the variation in outcomes, we hypothesized that interactions between risk factors may create phenotypes that experience differential benefits from CABG. Ultimately, our analysis indicated no significant interaction between phenogroup and the effect of CABG in addition to optimal medical therapy. Given the different risk profiles of the phenogroups, this implies that the long‐term benefits of CABG on all‐cause mortality, CV mortality, and a composite of all‐cause mortality and CV hospitalization are not modified by various constellations of risk factors determining a patient's underlying long‐term clinical risk, supporting current selection criteria for CABG in HFREF [19].

We identified four phenogroups within the STICHES cohort. Phenogroup 4 contained the highest proportion of US/Canada patients and was at the highest risk of all outcomes. This phenogroup was clinically characterized by the highest age, highest BMI, lowest exercise tolerance, highest burden of hypertension, diabetes, and triple‐vessel CAD, and poorest renal function. Phenogroup 4 appeared to represent a cardio‐renal‐metabolic phenotype which may reflect the presence of a higher‐risk state of concomitant dysglycemia, renal impairment, and atherosclerotic CV disease [20, 21]. Phenogroup 3 exhibited relatively lower hazard ratios of all outcomes compared to phenogroup 4. This phenogroup was clinically characterized by the highest rate of atrial fibrillation/flutter, intermediate rates of diabetes and renal insufficiency, and the poorest diastolic function, highest filling pressures, and lowest LVEF by echocardiography.

Perceived physical health status between phenogroups as measured by the Physical Composite Scale (PCS‐12) scores mirrored their relative clinical risk profiles. The lower‐risk phenogroups 1 and 2 exhibited the highest PCS‐12 scores, the intermediate‐risk phenogroup 3 exhibited intermediate scores, and the high‐risk phenogroup 4 exhibited the lowest scores. By contrast, perceived mental health status between phenogroups as measured by the Mental Composite Scale (MCS‐12) did not correlate well with their differing relative clinical risks. Within each phenogroup, neither perceived physical nor mental health status differed between treatment groups. The overall analysis of STICH quality‐of‐life data found small positive differences in PCS‐12 and MCS‐12 scores favoring patients receiving CABG at all time points; however, these differences were only consistently significant across time points for MCS‐12 scores [22]. Given the small effect size seen in the overall analysis, our phenogroups may not have been sufficiently sized to adequately power our within‐phenogroup analyses.

Markers of renal insufficiency (creatinine, BUN) were among the most discriminatory variables in the model, and clinically worse values were a key feature of the highest‐risk phenogroup 4. This result is consistent with prior clustering analyses in which the cardiorenal phenotype (i.e., concomitant cardiac and renal dysfunction) has been highlighted as a key driver of heightened risk for adverse cardiovascular outcomes [3, 12]. We note that our phenomapping analysis focuses on relationships between group characteristics, and that the discriminative impact of renal insufficiency must be considered in the context of the other characteristics comprising our phenogroups. In other words, when phenogrouping a datapoint, it is possible for poor renal function to be offset by low‐risk values of other variables (e.g., no diabetes, no smoking), resulting in a lower‐risk phenogroup assignment and an improved predicted long‐term prognosis.

Our study has several strengths. We utilized the large, robustly‐curated STICHES data set and further filtered clinical variables for high missingness and collinearity. We utilized a unique model‐based clustering method that allowed for integration of continuous and categorical variables, maximizing our coverage of potentially discriminative variables. We validated our findings internally within a randomly‐selected subset of the STICHES data set. Notable limitations of our study include an inability to externally validate our model as there are no datasets comparable to the STICHES cohort. The generalizability of our model is subject to the limitations of the STICHES data set, in which women and Black patients were underrepresented. The reduction in sample size caused by splitting the STICHES cohort into derivation and validation datasets limits the power of between‐ and within‐phenogroup statistical testing. Follow‐up for STICHES occurred between 2002 and 2015 in an era without sodium‐glucose transporter 2 inhibitor or angiotensin receptor blocker‐neprilysin inhibitor therapies for heart failure. However, eligibility criteria do not overlap between STICHES and clinical trials testing the efficacy of these newer agents. Furthermore, coronary revascularization for cardiomyopathies caused by ischemia is still a common clinical practice and recent data from the Revascularization for Ischemic Ventricular Dysfunction (REVIVED) trial are not conclusive [23, 24]. Finally, a fundamental limitation of high‐dimensional cluster analysis is that the complexity of the generated phenogroups limits the ability to statistically test for specific between‐group differences without multiple comparisons problems. In light of these limitations, we believe these results should be viewed as a validation of the broad efficacy of CABG and as hypothesis‐generating with regard to long‐term heart failure prognostic indices rather than as a proposal of a new heart failure risk scoring model.

## 5 Conclusion

We leveraged novel model‐based clustering in a cohort with HFREF and obstructive CAD to generate novel phenogroups with different long‐term risk profiles. We demonstrated that the impact of CABG versus medical therapy on long‐term outcomes did not differ between phenogroups, indicating that clinical heterogeneity in this population does not impact the benefit of CABG.

## Disclosure

Dr. Hendren reports research grant support from TriCog Health and the American Heart Association; and consulting services from Hendren LLC and Tosoh Inc. Dr. Grodin reports consulting fees from Pfizer, Eidos/BridgeBio, Alnylam, Alexion, Intellia, and Astra‐Zeneca. Dr. Farr reports consulting support from TransMedics. The remaining authors have no disclosures.

## Ethics Statement

This study was conducted on fully deidentified data publicly available at the National Heart, Lung, and Blood Institute's Biologic Specimen and Data Repository Information Coordinating Center, and as such, IRB review and informed consent were waived.

## Supporting information

Supporting information.

## Data Availability

The authors have nothing to report.
