# Supplementary material for: Phenomapping the Response of Patients With Ischemic Cardiomyopathy With Reduced Ejection Fraction to Surgical Revascularization
Source: Clin Cardiol. 2025 Feb 3;48(2):e70094. doi: 10.1002/clc.70094 (PMC11790598; doi:10.1002/clc.70094)

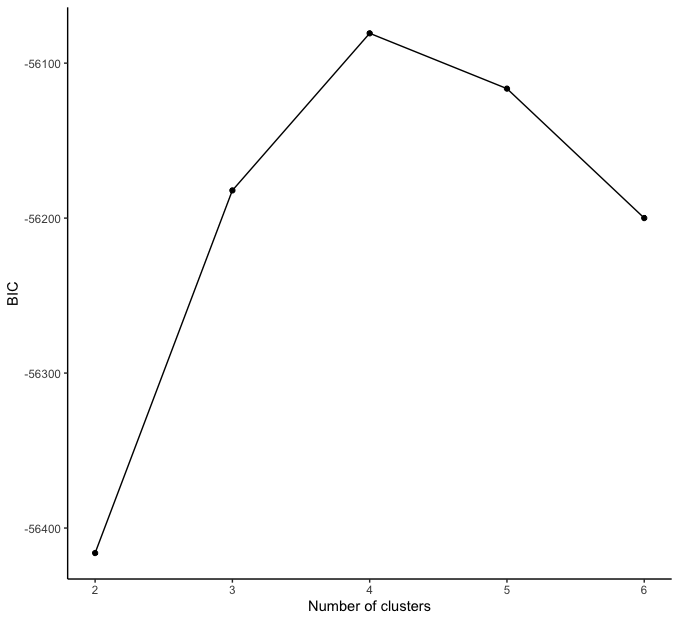


**Online Figure 1.** Bayesian Information Criteria (BIC) results for mixture model fit to STICHES data versus number of clusters.

**Online Table 1.** A list of all 58 variables used in the final model.

Age
Sex

Race

Body mass index

Systolic blood pressure

Pulse

Medical history: myocardial infarction, stroke, coronary artery bypass grafting, percutaneous coronary intervention, pacemaker*, implantable cardioverter-defibrillator, atrial fibrillation/atrial flutter, smoking, diabetes, hypertension, hyperlipidemia, renal insufficiency, peripheral vascular disease, cancer, depression

Baseline medications: angiotensin converting enzyme inhibitor, angiotensin receptor blocker, amiodarone, other antiarrhythmic, aspirin, clopidogrel, beta-blocker, digoxin, nitrate, insulin, statin, warfarin

Lab values: Hemoglobin, creatinine, sodium, blood urea nitrogen

New York Heart Association class

Canadian Cardiovascular Society angina class

Six-minute walk test distance

Duke coronary artery disease Index

Maximum degree of stenosis in left main, proximal left anterior descending, distal left anterior descending, left circumflex, and right coronary artery

Left ventricular ejection fraction

Left ventricular end-systolic volume

Mitral valve E velocity

Mitral regurgitation severity grade

E/A ratio

Right ventricular function

Left atrial area

Wall motion severity index

*Pacemakers were adjudicated to have been placed primarily for rate or re-synchronization purposes


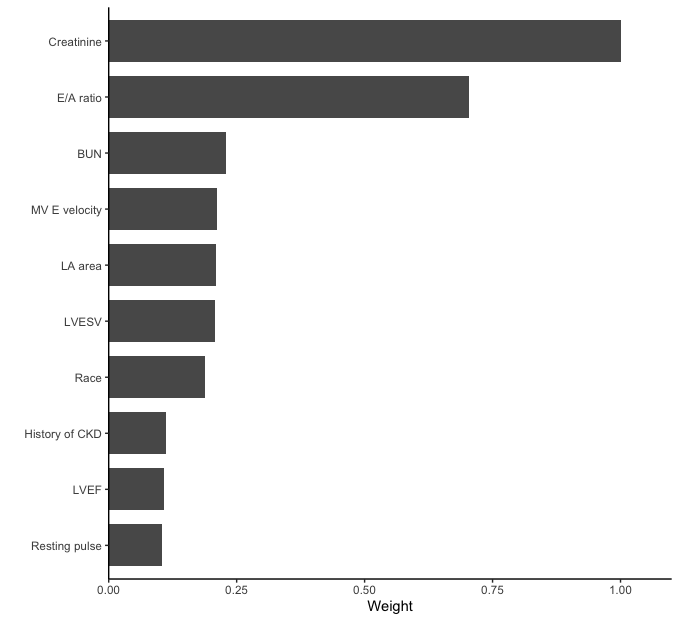

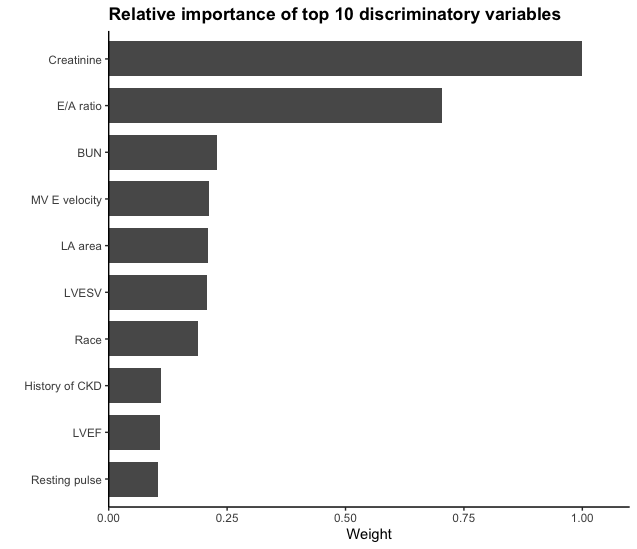


**Online Figure 2: Discriminatory variables in mixture model**

Top 10 most discriminatory variables in the best-fit mixture model. Variable importance is scaled to most discriminatory variable.

Abbreviations: BUN – blood urea nitrogen, MV – mitral valve, LA – left atrium, LVESV – left ventricular end systolic volume, CKD – chronic kidney disease, LVEF – left ventricular ejection fraction

**Online Table 2.** Baseline characteristics of patients in the STICHES trial stratified by derived phenogroup and treatment.

|  | Cluster 1 (n = 287) | | | Cluster 2 (n = 179) | | | Cluster 3 (n = 222) | | | Cluster 4 (n = 65) | | |
| --- | --- | --- | --- | --- | --- | --- | --- | --- | --- | --- | --- | --- |
|  | MED | CABG | p | MED | CABG | p | MED | CABG | p | MED | CABG | p |
| n | 138 (48.1) | 149 (51.9) | - | 86 (48.0) | 93 (52.0) | - | 102 (45.9) | 120 (54.1) | - | 32 (49.2) | 33 (50.8) | - |
| **Patient characteristics** |  |  |  |  |  |  |  |  |  |  |  |  |
| Mean age [years] (SD)* | 60.5 (8.9) | 60.9 (8.7) | 0.608 | 57.2 (8.2) | 58.0 (8.6) | 0.424 | 59.9 (9.1) | 60.1 (8.6) | 0.729 | 64.4 (11.2) | 65.3 (9.7) | 0.665 |
| Female sex (%)* | 18 (13.0) | 24 (16.1) | 0.507 | 13 (15.1) | 14 (15.1) | 1 | 7 (6.9) | 2 (1.7) | 0.084 | 5 (15.6) | 7 (21.2) | 0.751 |
| Race (%)* |  |  | 0.963 |  |  | 0.51 |  |  | 0.37 |  |  | 0.201 |
| White | 109 (79.0) | 117 (78.5) |  | 34 (39.5) | 32 (34.4) |  | 78 (76.5) | 80 (66.7) |  | 26 (81.2) | 25 (75.8) |  |
| Asian | 4 (2.9) | 4 (2.7) |  | 49 (57.0) | 60 (64.5) |  | 4 (3.9) | 7 (5.8) |  | 5 (15.6) | 2 (6.1) |  |
| Black | 3 (2.2) | 5 (3.4) |  | 2 (2.3) | 1 (1.1) |  | 1 (1.0) | 4 (3.3) |  | 1 (3.1) | 3 (9.1) |  |
| Other | 22 (15.9) | 23 (15.4) |  | 1 (1.2) | 0 (0.0) |  | 19 (18.6) | 29 (24.2) |  | 0 (0.0) | 3 (9.1) |  |
| Region (%) |  |  | 0.408 |  |  | 0.979 |  |  | 0.803 |  |  | 0.191 |
| North America | 23 (16.7) | 26 (17.4) |  | 13 (15.1) | 13 (14.0) |  | 26 (25.5) | 29 (24.2) |  | 17 (53.1) | 19 (57.6) |  |
| Europe | 106 (76.8) | 105 (70.5) |  | 0 (0.0) | 0 (0.0) |  | 0 (0.0) | 0 (0.0) |  | 0 (0.0) | 0 (0.0) |  |
| Asia-Pacific | 4 (2.9) | 10 (6.7) |  | 19 (22.1) | 19 (20.4) |  | 62 (60.8) | 73 (60.8) |  | 9 (28.1) | 9 (27.3) |  |
| South America | 5 (3.6) | 8 (5.4) |  | 51 (59.3) | 57 (61.3) |  | 7 (6.9) | 12 (10.0) |  | 6 (18.8) | 2 (6.1) |  |
| **Medical history** |  |  |  |  |  |  |  |  |  |  |  |  |
| Myocardial infarction (%)* | 121 (87.7) | 119 (79.9) | 0.081 | 57 (66.3) | 61 (65.6) | 1 | 78 (76.5) | 97 (80.8) | 0.51 | 27 (84.4) | 26 (78.8) | 0.751 |
| Percutaneous coronary intervention (%)* | 16 (11.6) | 17 (11.4) | 1 | 12 (14.0) | 12 (12.9) | 1 | 14 (13.7) | 22 (18.3) | 0.368 | 5 (15.6) | 5 (15.2) | 1 |
| Coronary artery bypass graft (%)* | 5 (3.6) | 6 (4.0) | 1 | 1 (1.2) | 0 (0.0) | 0.48 | 4 (3.9) | 7 (5.8) | 0.554 | 1 (3.1) | 3 (9.1) | 0.613 |
| Implantable cardioverter-defibrillator (%)* | 1 (0.7) | 0 (0.0) | 0.481 | 2 (2.3) | 3 (3.2) | 1 | 5 (4.9) | 5 (4.2) | 1 | 1 (3.1) | 6 (18.2) | 0.105 |
| Diabetes (%)* | 38 (27.5) | 49 (32.9) | 0.369 | 47 (54.7) | 50 (53.8) | 1 | 41 (40.2) | 32 (26.7) | 0.044 | 24 (75.0) | 26 (78.8) | 0.775 |
| Hypertension (%)* | 94 (68.1) | 97 (65.1) | 0.618 | 45 (52.3) | 50 (53.8) | 0.882 | 57 (55.9) | 66 (55.0) | 1 | 22 (68.8) | 24 (72.7) | 0.789 |
| Hyperlipidemia (%)* | 101 (73.2) | 94 (63.1) | 0.077 | 33 (38.8) | 34 (36.6) | 0.76 | 65 (63.7) | 77 (64.7) | 0.889 | 19 (59.4) | 25 (75.8) | 0.191 |
| Atrial flutter/atrial fibrillation (%)* | 6 (4.3) | 6 (4.0) | 1 | 5 (5.8) | 5 (5.4) | 1 | 26 (25.5) | 30 (25.0) | 1 | 5 (15.6) | 8 (24.2) | 0.537 |
| Chronic kidney disease (%)* | 5 (3.6) | 5 (3.4) | 1 | 2 (2.3) | 3 (3.2) | 1 | 1 (1.0) | 3 (2.5) | 0.626 | 19 (59.4) | 23 (69.7) | 0.443 |
| Active smoker (%)* | 23 (16.8) | 40 (26.8) | 0.046 | 13 (15.1) | 20 (21.5) | 0.336 | 35 (34.3) | 27 (22.5) | 0.053 | 3 (9.4) | 6 (18.2) | 0.475 |
| **Clinical characteristics** |  |  |  |  |  |  |  |  |  |  |  |  |
| NYHA stage 3+ (%)*^a^ | 43 (31.2) | 51 (34.2) | 0.669 | 22 (25.6) | 25 (26.9) | 0.978 | 50 (49.0) | 54 (45.0) | 0.643 | 18 (56.2) | 18 (54.5) | 1 |
| Mean pulse [bpm] (SD)* | 70.1 (10.7) | 71.6 (10.8) | 0.259 | 78.2 (10.5) | 79.5 (10.9) | 0.318 | 76.1 (14.0) | 75.9 (14.2) | 0.616 | 80.3 (26.8) | 79.3 (28.5) | 0.655 |
| Mean systolic blood pressure [mmHg] (SD)* | 124.8 (15.2) | 123.6 (15.0) | 0.389 | 122.5 (19.6) | 123.3 (18.7) | 0.969 | 116.4 (15.1) | 117.6 (16.2) | 0.937 | 116.4 (14.7) | 119.6 (22.1) | 0.712 |
| Mean diastolic blood pressure [mmHg] (SD) | 77.2 (10.4) | 76.6 (9.7) | 0.786 | 75.9 (10.6) | 78.5 (11.4) | 0.188 | 74.6 (9.8) | 74.6 (9.4) | 0.642 | 68.8 (12.7) | 68.2 (14.4) | 0.74 |
| Mean body mass index (SD)* | 28.3 (4.7) | 28.7 (4.3) | 0.332 | 24.9 (3.9) | 24.6 (3.8) | 0.692 | 26.9 (3.7) | 27.2 (4.0) | 0.693 | 28.6 (6.4) | 28.7 (6.2) | 0.927 |
| Six-minute walk test distance [m] (SD)* | 343.7 (111.7) | 341.2 (102.7) | 0.932 | 329.4 (131.3) | 322.6 (117.6) | 0.933 | 338.9 (114.9) | 351.0 (133.5) | 0.454 | 248.1 (87.2) | 267.0 (145.4) | 0.694 |
| **Baseline medications** |  |  |  |  |  |  |  |  |  |  |  |  |
| Aspirin (%)* | 124 (89.9) | 126 (84.6) | 0.218 | 75 (87.2) | 76 (81.7) | 0.411 | 79 (77.5) | 96 (80.0) | 0.742 | 28 (87.5) | 32 (97.0) | 0.197 |
| Clopidogrel (%)* | 8 (5.8) | 15 (10.1) | 0.199 | 37 (43.0) | 41 (44.1) | 1 | 9 (8.8) | 13 (10.8) | 0.659 | 11 (34.4) | 3 (9.1) | 0.017 |
| Statin (%)* | 119 (86.2) | 110 (73.8) | 0.012 | 78 (90.7) | 83 (89.2) | 0.807 | 78 (76.5) | 95 (79.2) | 0.631 | 24 (75.0) | 26 (78.8) | 0.775 |
| Angiotensin-converting enzyme inhibitor (%)* | 112 (81.2) | 127 (85.2) | 0.429 | 69 (80.2) | 76 (81.7) | 0.85 | 85 (83.3) | 105 (87.5) | 0.445 | 21 (65.6) | 22 (66.7) | 1 |
| Angiotensin receptor blocker (%)* | 12 (8.7) | 12 (8.1) | 1 | 10 (11.6) | 8 (8.6) | 0.621 | 12 (11.8) | 7 (5.8) | 0.149 | 4 (12.5) | 8 (24.2) | 0.339 |
| Beta-blocker (%)* | 132 (95.7) | 128 (85.9) | 0.005 | 70 (81.4) | 76 (81.7) | 1 | 90 (88.2) | 101 (84.2) | 0.44 | 28 (87.5) | 27 (81.8) | 0.733 |
| K-wasting diuretic (%)* | 77 (55.8) | 98 (65.8) | 0.091 | 54 (62.8) | 62 (66.7) | 0.64 | 73 (71.6) | 79 (66.4) | 0.467 | 29 (90.6) | 29 (87.9) | 1 |
| K-sparing diuretic (%)* | 61 (44.2) | 66 (44.3) | 1 | 34 (39.5) | 46 (49.5) | 0.229 | 57 (55.9) | 66 (55.0) | 1 | 12 (37.5) | 14 (42.4) | 0.801 |
| Digoxin (%)* | 18 (13.0) | 15 (10.1) | 0.463 | 20 (23.3) | 29 (31.2) | 0.246 | 25 (24.5) | 23 (19.2) | 0.414 | 16 (50.0) | 9 (27.3) | 0.077 |
| Nitrate (%)* | 72 (52.2) | 89 (59.7) | 0.234 | 54 (62.8) | 59 (63.4) | 1 | 42 (41.2) | 48 (40.3) | 1 | 14 (43.8) | 18 (54.5) | 0.46 |
| Warfarin (%)* | 12 (8.7) | 9 (6.0) | 0.497 | 7 (8.1) | 2 (2.2) | 0.09 | 21 (20.6) | 13 (10.8) | 0.061 | 5 (15.6) | 5 (15.2) | 1 |
| Amiodarone (%)* | 10 (7.2) | 10 (6.7) | 1 | 10 (11.6) | 8 (8.6) | 0.621 | 8 (7.8) | 16 (13.3) | 0.202 | 9 (28.1) | 4 (12.1) | 0.13 |
| **Laboratory measurements** |  |  |  |  |  |  |  |  |  |  |  |  |
| Mean sodium [mEq/L] (SD)* | 140.1 (3.0) | 139.9 (3.0) | 0.642 | 138.3 (4.9) | 137.5 (6.3) | 0.335 | 139.8 (3.1) | 140.1 (3.0) | 0.412 | 137.2 (4.3) | 136.7 (4.3) | 0.483 |
| Mean creatinine [mg/dL] (SD)* | 1.1 (0.2) | 1.1 (0.2) | 0.762 | 1.1 (0.2) | 1.1 (0.2) | 0.873 | 1.1 (0.2) | 1.1 (0.2) | 0.877 | 2.2 (1.3) | 2.4 (2.7) | 0.331 |
| Mean blood urea nitrogen [mg/dL] (SD)* | 26.8 (18.1) | 30.3 (18.5) | 0.065 | 22.4 (9.3) | 20.2 (10.6) | 0.053 | 31.3 (17.2) | 34.0 (20.7) | 0.745 | 50.9 (30.4) | 61.0 (53.1) | 0.619 |
| Mean hemoglobin [g/dL] (SD)* | 14.4 (1.4) | 14.1 (1.5) | 0.109 | 13.2 (1.8) | 12.8 (1.8) | 0.209 | 14.2 (1.9) | 14.1 (1.5) | 0.919 | 12.3 (1.9) | 12.1 (1.5) | 0.788 |
| **Left heart catheterization characteristics** |  |  |  |  |  |  |  |  |  |  |  |  |
| Mean Duke CAD index (SD)* | 59.2 (20.7) | 58.1 (20.1) | 0.655 | 57.9 (20.1) | 58.3 (21.4) | 0.964 | 60.2 (23.5) | 60.1 (21.1) | 0.902 | 64.1 (21.4) | 65.8 (22.8) | 0.549 |
| >50% left main stenosis (%)*^b^ | 2 (1.4) | 3 (2.0) | 1 | 1 (1.2) | 2 (2.2) | 1 | 5 (4.9) | 3 (2.5) | 0.551 | 2 (6.2) | 4 (12.1) | 0.697 |
| >75% proximal LAD stenosis (%)*^b^ | 97 (70.3) | 112 (75.2) | 0.426 | 65 (75.6) | 64 (68.8) | 0.4 | 68 (66.7) | 77 (64.2) | 0.804 | 18 (56.2) | 19 (57.6) | 1 |
| >75% distal LAD stenosis (%)*^b^ | 37 (27.6) | 31 (20.8) | 0.231 | 21 (24.4) | 31 (33.3) | 0.251 | 32 (31.7) | 33 (27.5) | 0.595 | 16 (50.0) | 15 (45.5) | 0.906 |
| >75% left circumflex stenosis (%)*^b^ | 75 (54.3) | 71 (47.7) | 0.31 | 50 (58.1) | 50 (53.8) | 0.661 | 59 (57.8) | 77 (64.2) | 0.409 | 24 (75.0) | 25 (75.8) | 1 |
| >75% right coronary stenosis (%)*^b^ | 88 (63.8) | 99 (66.4) | 0.725 | 55 (64.0) | 65 (69.9) | 0.493 | 73 (71.6) | 85 (70.8) | 1 | 26 (81.2) | 25 (75.8) | 0.813 |
| Number of vessels with >75% stenosis (%) |  |  | 0.92 |  |  | 0.364 |  |  | 0.366 |  |  | 1 |
| 0 | 3 (2.2) | 4 (2.7) |  | 0 (0.0) | 1 (1.1) |  | 2 (2.0) | 4 (3.3) |  | 0 (0.0) | 0 (0.0) |  |
| 1 | 35 (25.4) | 37 (24.8) |  | 17 (19.8) | 22 (23.7) |  | 26 (25.5) | 21 (17.5) |  | 6 (18.8) | 5 (15.2) |  |
| 2 | 54 (39.1) | 63 (42.3) |  | 43 (50.0) | 36 (38.7) |  | 32 (31.4) | 48 (40.0) |  | 7 (21.9) | 8 (24.2) |  |
| 3 | 46 (33.3) | 45 (30.2) |  | 26 (30.2) | 34 (36.6) |  | 42 (41.2) | 47 (39.2) |  | 19 (59.4) | 20 (60.6) |  |
| **Echocardiographic characteristics** |  |  |  |  |  |  |  |  |  |  |  |  |
| Mean ejection fraction [%] (SD)* | 29.9 (9.6) | 28.2 (8.0) | 0.279 | 33.4 (7.8) | 33.4 (8.4) | 0.762 | 22.8 (6.3) | 22.9 (6.2) | 0.846 | 25.9 (6.7) | 26.3 (6.0) | 0.406 |
| Mean LV end-systolic volume [mL] (SD)* | 160.3 (62.5) | 157.4 (55.2) | 0.886 | 117.5 (31.8) | 103.9 (36.7) | 0.028 | 224.7 (75.9) | 203.9 (59.9) | 0.045 | 160.9 (43.6) | 151.7 (52.5) | 0.318 |
| Mean LV end-diastolic volume [mL] (SD) | 224.2 (69.0) | 215.0 (60.4) | 0.376 | 176.0 (41.1) | 156.0 (39.7) | 0.014 | 287.9 (81.3) | 263.3 (67.7) | 0.037 | 216.6 (47.2) | 205.6 (64.4) | 0.264 |
| Mean stroke volume [mL] (SD) | 73.9 (23.2) | 64.2 (16.4) | 0.007 | 64.9 (17.1) | 59.0 (18.1) | 0.143 | 58.0 (17.9) | 57.7 (20.1) | 0.675 | 57.8 (21.8) | 63.4 (19.5) | 0.342 |
| Mean MV E velocity [m/s] (SD)* | 0.5 (0.1) | 0.5 (0.2) | 0.88 | 0.8 (0.2) | 0.8 (0.3) | 0.675 | 0.9 (0.2) | 0.8 (0.2) | 0.016 | 0.8 (0.3) | 0.8 (0.3) | 0.789 |
| Mean MV A velocity [m/s] (SD) | 0.8 (0.1) | 0.8 (0.2) | 0.774 | 0.7 (0.2) | 0.6 (0.2) | 0.406 | 0.5 (0.2) | 0.4 (0.2) | 0.024 | 0.6 (0.2) | 0.7 (0.3) | 0.363 |
| Mean mitral annular e’ velocity [m/s] (SD)^†^ | 0.1 (0.0) | 0.0 (0.0) | 0.198 | 0.1 (0.0) | 0.1 (0.0) | 0.881 | 0.1 (0.0) | 0.1 (0.0) | 0.924 | 0.0 (0.0) | 0.1 (0.0) | 0.22 |
| Mean E/A ratio (SD)* | 0.7 (0.2) | 0.7 (0.2) | 0.97 | 1.4 (0.7) | 1.6 (1.0) | 0.492 | 2.1 (1.1) | 2.3 (1.4) | 0.463 | 1.6 (1.0) | 1.5 (0.9) | 0.56 |
| Mean E/e’ ratio (SD) | 14.5 (8.7) | 14.3 (7.4) | 0.688 | 20.2 (8.9) | 19.8 (11.0) | 0.643 | 25.1 (10.3) | 21.5 (10.1) | 0.07 | 24.2 (9.9) | 21.8 (7.4) | 0.518 |
| Mitral regurgitation severity grade 3+ (%)*^c^ | 2 (1.7) | 1 (0.7) | 0.9 | 3 (4.2) | 5 (7.1) | 0.7 | 22 (23.7) | 19 (19.6) | 0.614 | 4 (12.9) | 0 (0.0) | 0.157 |
| Moderate-severe RV dysfunction (%)*^d^ | 2 (1.6) | 6 (4.5) | 0.334 | 3 (4.1) | 3 (4.1) | 1 | 25 (27.8) | 35 (34.0) | 0.44 | 7 (22.6) | 4 (15.4) | 0.727 |
| Mean LA area [cm^2^] (SD)* | 21.4 (4.6) | 21.0 (4.1) | 0.632 | 20.1 (3.6) | 18.8 (3.8) | 0.09 | 27.5 (5.0) | 27.2 (5.3) | 0.743 | 23.5 (4.7) | 23.5 (3.9) | 0.93 |
| Mean LA volume [mL] (SD) | 71.0 (20.9) | 68.6 (19.4) | 0.578 | 68.6 (18.8) | 59.9 (18.6) | 0.048 | 105.3 (30.1) | 98.9 (26.6) | 0.174 | 79.7 (21.2) | 83.9 (18.0) | 0.389 |
| Mean posterior wall thickness [cm] (SD) | 1.1 (0.2) | 1.0 (0.1) | 0.356 | 0.9 (0.2) | 0.6 (0.1) | 0.094 | 0.9 (0.2) | 0.8 (0.3) | 0.858 | 1.0 (0.2) | 0.9 (0.2) | 0.417 |
| Mean interventricular septum thickness [cm] (SD) | 1.2 (0.2) | 0.9 (0.3) | 0.068 | 0.8 (0.2) | 0.8 (0.2) | 0.664 | 0.8 (0.2) | 1.0 (0.1) | 0.123 | 1.1 (0.1) | 0.9 (0.1) | 0.057 |
| Mean sphericity index (SD) | 1.5 (0.2) | 1.5 (0.2) | 0.663 | 1.5 (0.2) | 1.5 (0.2) | 0.59 | 1.4 (0.2) | 1.4 (0.2) | 0.841 | 1.4 (0.1) | 1.6 (0.3) | 0.018 |
| Mean wall motion severity index (SD)* | 2.3 (0.3) | 2.2 (0.3) | 0.156 | 2.1 (0.3) | 2.1 (0.3) | 0.942 | 2.3 (0.3) | 2.4 (0.3) | 0.032 | 2.3 (0.3) | 2.3 (0.4) | 0.953 |

Abbreviations: NYHA – New York Heart Association, CCS – Canadian Cardiovascular Society, LA – left atrium, LV – left ventricle, RV – right ventricle, CAD – coronary artery disease, LAD – left anterior descending, MED – optimal medical therapy, CABG – optimal medical therapy in addition to coronary artery bypass grafting

*Variable was part of final mixture model after missingness and collinearity check steps

^a^In full model, NYHA class was an ordinal variable with levels 1, 2, 3, 4

^b^In full model, vessel stenosis was an ordinal variable with levels 0-24%, 25-49%, 50-74%, 75-94%, and 95-100%

^c^In full model, mitral regurgitation was an ordinal variable with levels 0, 1, 2, 3, 4

^d^In full model, RV dysfunction was an ordinal variable with levels normal, mild, moderate, severe

†Average of septal and lateral mitral annular e’ velocities

**Online Table 3. Baseline angiographic and echocardiographic characteristics**

Baseline angiographic and echocardiographic characteristics of patients in the STICHES trial stratified by model-based clustering derived phenogroup.

|  | Cluster 1 | Cluster 2 | Cluster 3 | Cluster 4 | p-value |
| --- | --- | --- | --- | --- | --- |
| n | 287 | 179 | 222 | 65 | - |
| **Left heart catheterization characteristics** |  |  |  |  |  |
| Mean Duke CAD index (SD)* | 58.6 (20.4) | 58.1 (20.7) | 60.2 (22.2) | 65.0 (22.0) | 0.105 |
| >50% left main stenosis (%)*^a^ | 5 (1.74) | 3 (1.68) | 8 (3.60) | 6 (9.23) | 0.008 |
| >75% proximal LAD stenosis (%)*^a^ | 209 (72.82) | 129 (72.07) | 145 (65.32) | 37 (56.92) | 0.035 |
| >75% distal LAD stenosis (%)*^a^ | 68 (24.03) | 52 (29.05) | 65 (29.41) | 31 (47.69) | 0.002 |
| >75% left circumflex stenosis (%)*^a^ | 146 (50.87) | 100 (55.87) | 136 (61.26) | 49 (75.38) | 0.002 |
| >75% right coronary stenosis (%)*^a^ | 187 (65.16) | 120 (67.04) | 158 (71.17) | 51 (78.46) | 0.145 |
| Number of vessels with >75% stenosis (%) |  |  |  |  | 0.003 |
| 0 | 7 (2.4) | 1 (0.6) | 6 (2.7) | 0 (0.0) | - |
| 1 | 72 (25.1) | 39 (21.8) | 47 (21.2) | 11 (16.9) | - |
| 2 | 117 (40.8) | 79 (44.1) | 80 (36.0) | 15 (23.1) | - |
| 3 | 91 (31.7) | 60 (33.5) | 89 (40.1) | 39 (60.0) | - |
| **Echocardiographic characteristics** |  |  |  |  |  |
| Mean ejection fraction [%] (SD)* | 29.0 (8.8) | 33.4 (8.1) | 22.9 (6.2) | 26.1 (6.3) | <0.001 |
| Mean LV end-systolic volume [mL] (SD)* | 158.8 (58.6) | 111.2 (34.6) | 213.5 (68.3) | 156.1 (48.2) | <0.001 |
| Mean LV end-diastolic volume [mL] (SD) | 219.3 (64.6) | 166.8 (41.5) | 274.6 (75.1) | 210.9 (56.5) | <0.001 |
| Mean stroke volume [mL] (SD) | 69.1 (20.7) | 61.7 (17.8) | 57.8 (18.9) | 60.7 (20.5) | <0.001 |
| Mean MV E velocity [m/s] (SD)* | 0.54 (0.15) | 0.83 (0.25) | 0.88 (0.22) | 0.85 (0.28) | <0.001 |
| Mean MV A velocity [m/s] (SD) | 0.79 (0.17) | 0.66 (0.24) | 0.49 (0.22) | 0.66 (0.26) | <0.001 |
| Mean mitral annular e’ velocity [m/s] (SD)^†^ | 0.05 (0.02) | 0.06 (0.02) | 0.05 (0.02) | 0.05 (0.02) | 0.048 |
| Mean E/A ratio (SD)* | 0.70 (0.19) | 1.47 (0.85) | 2.21 (1.26) | 1.56 (0.96) | <0.001 |
| Mean E/e’ ratio (SD) | 14.4 (8.0) | 20.0 (9.8) | 23.3 (10.3) | 22.8 (8.4) | <0.001 |
| Mitral regurgitation severity grade 3+ (%)*^b^ | 3 (1.20) | 8 (5.67) | 41 (21.58) | 4 (6.90) | <0.001 |
| Moderate-severe RV dysfunction (%)*^c^ | 8 (3.12) | 6 (4.11) | 60 (31.09) | 11 (19.30) | <0.001 |
| Mean LA area [cm^2^] (SD)* | 21.2 (4.3) | 19.4 (3.7) | 27.3 (5.2) | 23.5 (4.3) | <0.001 |
| Mean LA volume [mL] (SD) | 69.9 (20.2) | 64.2 (19.1) | 101.8 (28.3) | 81.8 (19.6) | <0.001 |
| Mean posterior wall thickness [cm] (SD) | 1.1 (0.2) | 0.8 (0.2) | 0.8 (0.3) | 1.0 (0.2) | 0.101 |
| Mean interventricular septum thickness [cm] (SD) | 1.0 (0.3) | 0.8 (0.2) | 0.9 (0.2) | 0.9 (0.2) | 0.367 |
| Mean sphericity index (SD) | 1.5 (0.2) | 1.5 (0.2) | 1.4 (0.2) | 1.5 (0.2) | <0.001 |
| Mean wall motion severity index (SD)* | 2.3 (0.3) | 2.1 (0.3) | 2.4 (0.3) | 2.3 (0.4) | <0.001 |

Abbreviations: LA – left atrium, LV – left ventricle, RV – right ventricle, CAD – coronary artery disease, LAD – left anterior descending

*Variable was part of final mixture model after missingness and collinearity check steps

^a^In full model, vessel stenosis was an ordinal variable with levels 0-24%, 25-49%, 50-74%, 75-94%, and 95-100%

^b^In full model, mitral regurgitation was an ordinal variable with levels 0, 1, 2, 3, 4

^c^In full model, RV dysfunction was an ordinal variable with levels normal, mild, moderate, severe

†Average of septal and lateral mitral annular e’ velocities

**Online Table 4.** **Cumulative incidence rates of key outcomes**

Cumulative incidence rates of key outcomes in the STICHES trial stratified by model-based clustering derived phenogroups.

|  | Cluster 1  (n = 287) | Cluster 2  (n = 179) | Cluster 3  (n = 222) | Cluster 4  (n = 65) |
| --- | --- | --- | --- | --- |
| All-cause mortality (%) | 156 (55.4) | 94 (52.5) | 165 (74.3) | 51 (78.4) |
| Cardiovascular mortality (%) | 107 (37.3) | 69 (38.5) | 127 (57.2) | 34 (52.3) |
| Death and cardiovascular hospitalization (%) | 232 (80.8) | 118 (65.9) | 200 (90.1) | 58 (89.2) |

**Online Table 5.** Post-coronary artery bypass grafting short-term outcomes in the STICHES trial stratified by derived phenogroup. Displayed p-values are the result of the Bonferroni-adjusted Kruskal-Wallis test (alpha = 0.01).

|  | Cluster 1 | Cluster 2 | Cluster 3 | Cluster 4 | p-value |
| --- | --- | --- | --- | --- | --- |
| Number of patients assigned to CABG arm | 149 | 93 | 120 | 33 | - |
| Mean post-surgery hospital stay length [days] (SD) | 11.9 (11.1) | 11.0 (12.1) | 11.8 (9.1) | 13.4 (10.6) | 0.13 |
| Hospitalization length > 30 days (%) | 5 (3.5) | 3 (3.6) | 4 (3.9) | 2 (6.5) | 0.86 |
| Death or not discharged 30 days after surgery (%) | 10 (6.7) | 6 (6.5) | 10 (8.3) | 5 (15.2) | 0.4 |
| Death within 30 days after surgery (%) | 5 (3.5) | 3 (3.6) | 6 (5.9) | 3 (9.7) | 0.41 |
| Myocardial infarction within 30 days after surgery (%) | 1 (0.7) | 0 (0.0) | 0 (0.0) | 0 (0.0) | 1 |
| Stroke within 30 days after surgery (%) | 3 (2.1) | 2 (2.4) | 3 (2.9) | 0 (0.0) | 0.95 |

|  | Cluster 1  (n = 183) | Cluster 2  (n = 105) | Cluster 3  (n = 127) | Cluster 4  (n = 44) |
| --- | --- | --- | --- | --- |
| All-cause mortality (%) | 100 (54.6) | 53 (50.5) | 98 (77.2) | 40 (90.9) |
| Cardiovascular mortality (%) | 59 (32.2) | 37 (35.2) | 83 (65.4) | 28 (63.6) |
| Death and cardiovascular hospitalization (%) | 145 (79.2) | 82 (78.1) | 115 (90.6) | 41 (93.2) |

**Online Table 6.** Cumulative incidence rates of key outcomes in the STICHES trial stratified by derived phenogroup in the internal validation cohort.

**Online Figure 3.** Kaplan-Meier curves of key outcomes in STICHES trial stratified by derived phenogroup in internal validation cohort. P-values in the lower left are the result of the log-rank test.


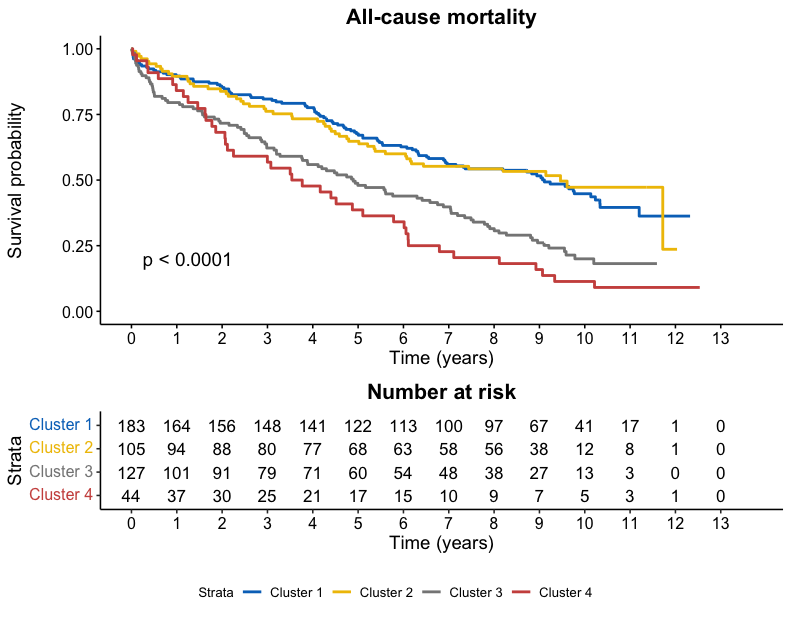


**
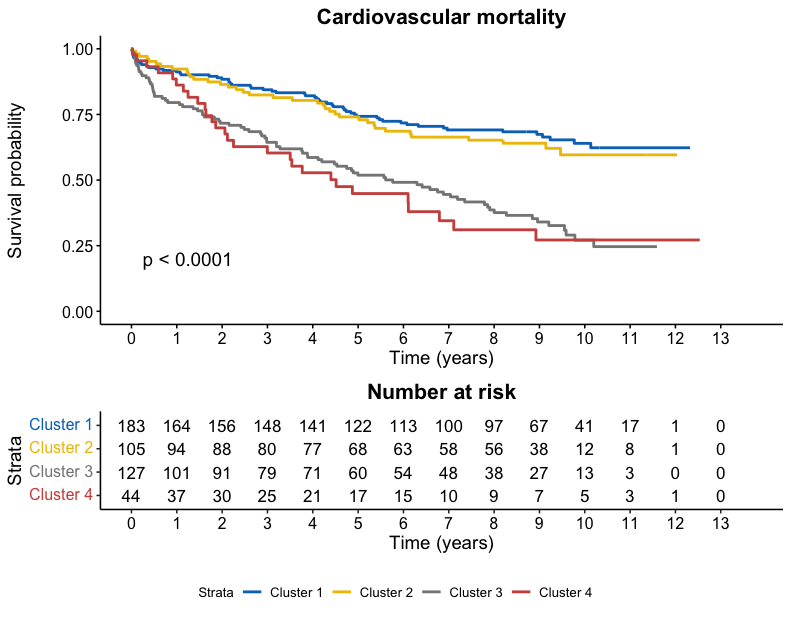
**


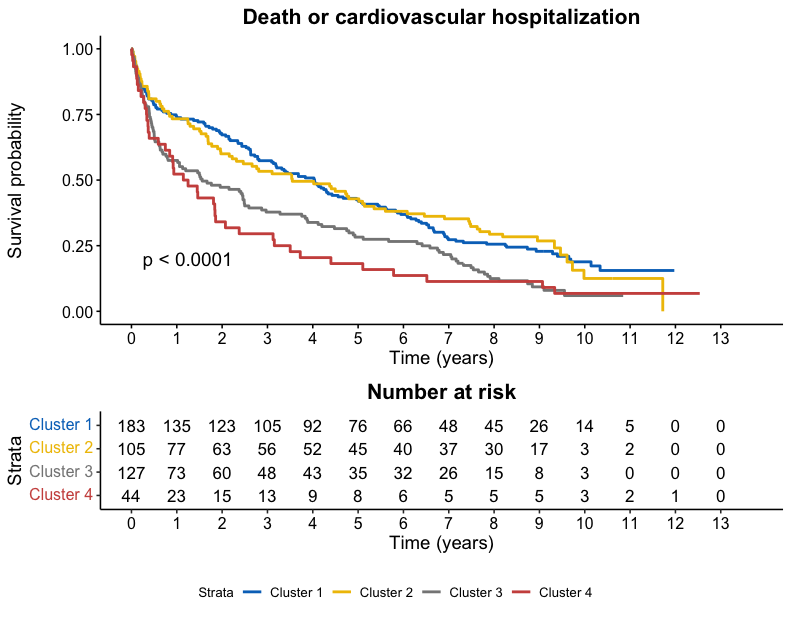


**Online Table 7.** Hazard ratios of key outcomes in the STICHES trial stratified by derived phenogroup in the internal validation dataset.

|  | Hazard ratio (95% CI) | p-value |
| --- | --- | --- |
| **All-cause mortality** |  |  |
| Cluster 1 | Ref. | N/A |
| Cluster 2 | 1.17 (0.75 – 1.82) | 0.50 |
| Cluster 3 | 1.75 (1.28 – 2.39) | <0.001 |
| Cluster 4 | 2.37 (1.54 – 3.66) | <0.001 |
| **Cardiovascular mortality** |  |  |
| Cluster 1 | Ref. | N/A |
| Cluster 2 | 1.23 (0.71 – 2.14) | 0.46 |
| Cluster 3 | 2.45 (1.69 – 3.55) | <0.001 |
| Cluster 4 | 2.78 (1.65 – 4.70) | <0.001 |
| **Death or cardiovascular hospitalization** |  |  |
| Cluster 1 | Ref. | N/A |
| Cluster 2 | 1.26 (0.88 – 1.80) | 0.21 |
| Cluster 3 | 1.31 (0.99 – 1.72) | 0.056 |
| Cluster 4 | 1.68 (1.10 – 2.56) | 0.015 |

**Online Figure 4. Quality of life measures between derived phenogroups**

12-Item Short Form Survey (SF-12) physical (PCS-12) and mental (MCS-12) component scores over the duration of the STICHES trial stratified by model-based clustering derived phenogroups. The SF-12 component population average of 50 is denoted by a dashed line. Intervals with significant (Bonferroni-adjusted ANOVA, alpha = 0.01) score differences between clusters are denoted by an asterisk.


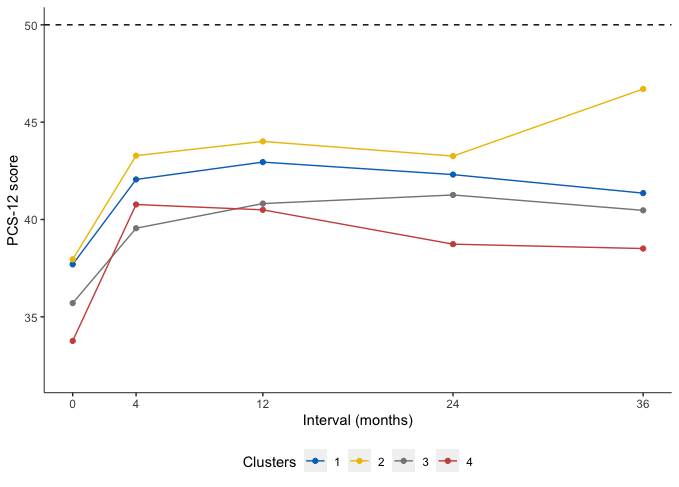


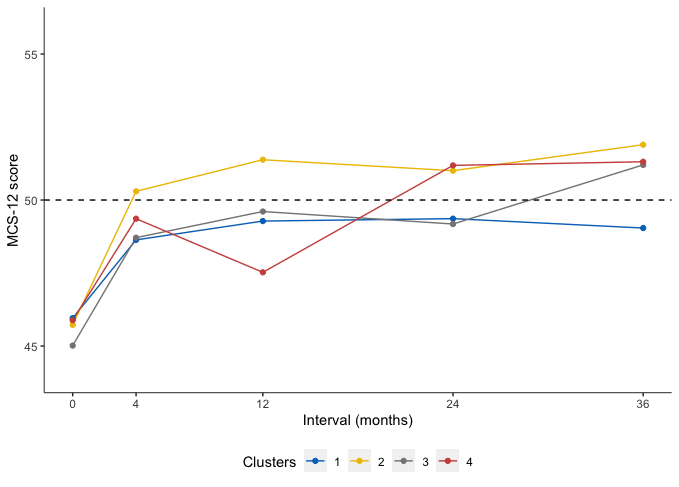


*****

*****

*****

*****

**Online Figure 5.** 12-Item Short Form Survey (SF-12) physical (PCS-12) and mental (MCS-12) component scores over the duration of the STICHES trial stratified by treatment within each derived phenogroup. The SF-12 component population average of 50 is denoted by a dashed line. There were no significant score differences between treatment groups in any cluster (Bonferroni-adjusted t-test, alpha = 0.0025).


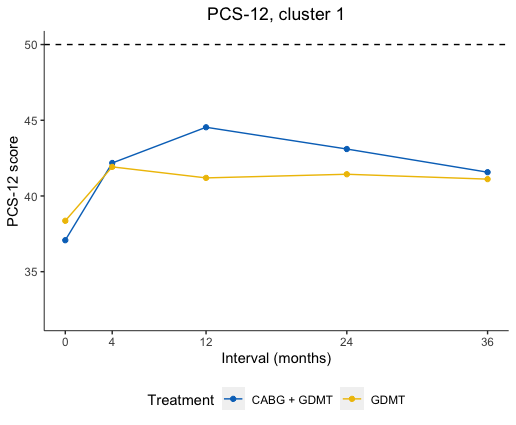

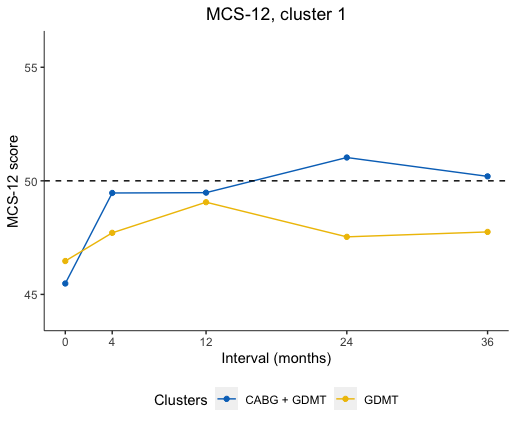

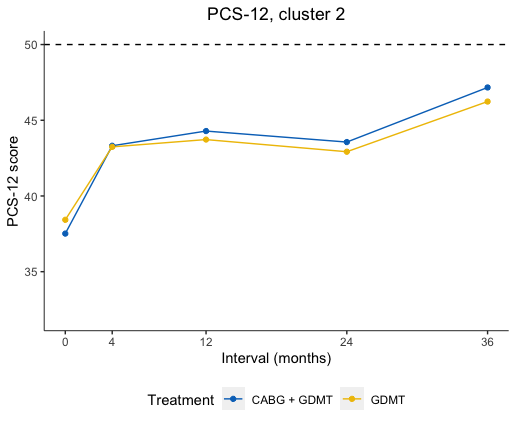

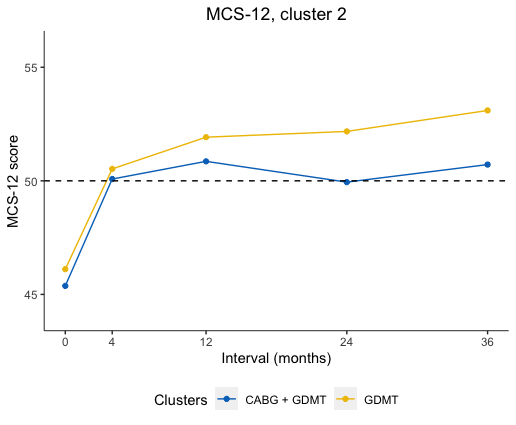

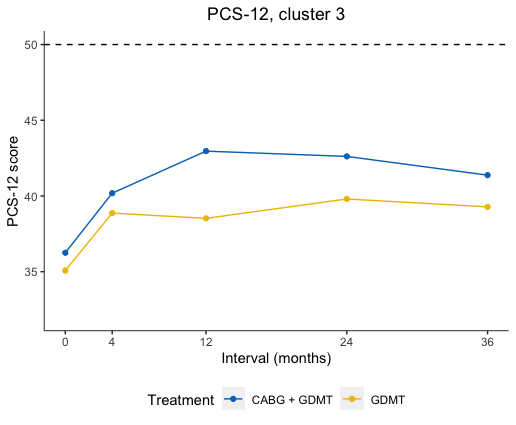

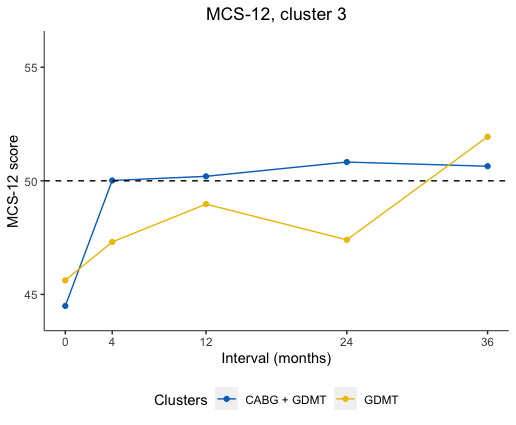


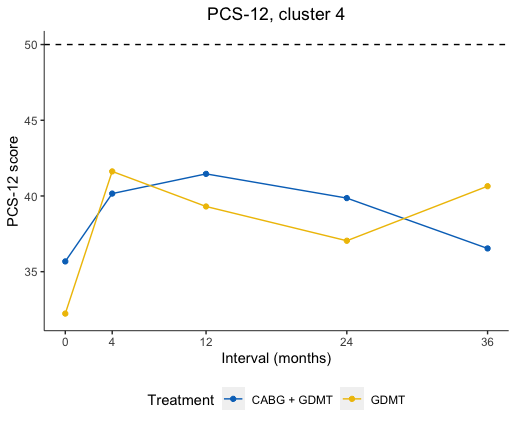

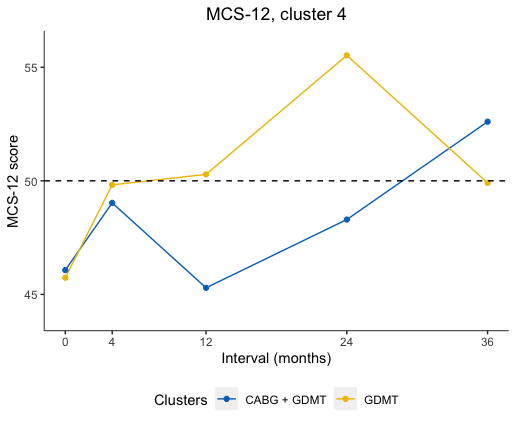

Supplement: Supplementary file 1 — Supporting information. [file CLC-48-e70094-s001.docx]
